# Evaluating diagnostic accuracy and determining optimal diagnostic thresholds of different approaches to [^68^Ga]-DOTATATE PET/MRI analysis in patients with meningioma

**DOI:** 10.1038/s41598-022-13467-9

**Published:** 2022-06-03

**Authors:** Sean H. Kim, Michelle Roytman, Gabriela Madera, Rajiv S. Magge, Benjamin Liechty, Rohan Ramakrishna, Susan C. Pannullo, Theodore H. Schwartz, Nicolas A. Karakatsanis, Joseph R. Osborne, Eaton Lin, Jonathan P. S. Knisely, Jana Ivanidze

**Affiliations:** 1 Department of Radiology, Weill Cornell Medicine, 525 E. 68th St., New York, NY 10021 USA; 2 Department of Neurology, Weill Cornell Medicine, 525 E. 68th St., Starr Pavilion 651, New York, NY 10021 USA; 3 Department of Pathology and Laboratory Medicine, Weill Cornell Medicine, 525 E. 68th St., Starr Pavilion, 10th Floor, New York, NY 10021 USA; 4 Department of Neurological Surgery, Weill Cornell Medicine, 1305 York Avenue, 9th Floor, New York, NY 10021 USA; 5 Department of Radiation Oncology, Stich Radiation Center, Weill Cornell Medicine, 525 E. 68th St., New York, NY 10065 USA

**Keywords:** Medical research, Diagnostic markers

## Abstract

Multiple approaches with [^68^Ga]-DOTATATE, a somatostatin analog PET radiotracer, have demonstrated clinical utility in evaluation of meningioma but have not been compared directly. Our purpose was to compare diagnostic performance of different approaches to quantitative brain [^68^Ga]-DOTATATE PET/MRI analysis in patients with suspected meningioma recurrence and to establish the optimal diagnostic threshold for each method. Patients with suspected meningioma were imaged prospectively with [^68^Ga]-DOTATATE brain PET/MRI. Lesions were classified as meningiomas and post-treatment change (PTC), using follow-up pathology and MRI as reference standard. Lesions were reclassified using the following methods: absolute maximum SUV threshold (SUV), SUV ratio (SUVR) to superior sagittal sinus (SSS) (SUVRsss), SUVR to the pituitary gland (SUVRpit), and SUVR to the normal brain parenchyma (SUVRnorm). Diagnostic performance of the four methods was compared using contingency tables and McNemar’s test. Previously published pre-determined thresholds were assessed where applicable. The optimal thresholds for each method were identified using Youden’s J statistics. 166 meningiomas and 41 PTC lesions were identified across 62 patients. SUV, SUVRsss, SUVRpit, and SUVRnorm of meningioma were significantly higher than those of PTC (P < 0.0001). The optimal thresholds for SUV, SUVRsss, SUVRpit, and SUVRnorm were 4.7, 3.2, 0.3, and 62.6, respectively. At the optimal thresholds, SUV had the highest specificity (97.6%) and SUVRsss had the highest sensitivity (86.1%). An ROC analysis of SUV, SUVRsss, SUVRpit, and SUVRnorm revealed AUC of 0.932, 0.910, 0.915, and 0.800, respectively (P < 0.0001). Developing a diagnostic threshold is key to wider clinical translation of [^68^Ga]-DOTATATE PET/MRI in meningioma evaluation. We found that the SUVRsss method may have the most robust combination of sensitivity and specificity in the diagnosis of meningioma in the post-treatment setting, with the optimal threshold of 3.2. Future studies validating our findings in different patient populations are needed to continue optimizing the diagnostic performance of [^68^Ga]-DOTATATE PET/MRI in meningioma patients.

**Trial registration**: ClinicalTrials.gov Identifier: NCT04081701. Registered 9 September 2019. https://clinicaltrials.gov/ct2/show/NCT04081701.

## Introduction

Meningiomas are the most common primary intracranial tumors, accounting for more than a third of all primary brain tumors^1^. While gross-total resection is the standard of care, approximately 34–50% of patients undergo subtotal resection which is associated with lower rates of progression-free survival (PFS) and overall survival (OS), necessitating subsequent active surveillance with serial imaging for detection of any residual or recurrent tumor^2^. In patients with high-risk meningioma, which include newly diagnosed or recurrent WHO grade 3 meningioma of any resection extent, a recurrent WHO grade 2 tumor of any resection extent, or a newly diagnosed WHO grade 2 tumor following subtotal resection, postoperative adjuvant radiotherapy (RT) is often pursued^3^. Magnetic resonance imaging (MRI) is the gold standard for the diagnosis and treatment planning of meningioma. However, especially in the post-surgical and post-RT setting, MRI can have suboptimal sensitivity and specificity in distinguishing meningioma from post-treatment scarring and inflammation^4^. MRI is also limited in cases of small lesion size, infiltrative or “en plaque” lesions, osseous or parenchymal invasion, and challenging locations such as the skull base and cavernous sinus^5–7^. Thus, more sensitive and specific imaging biomarkers have the potential to improve diagnosis, treatment, and thereby clinical outcomes in meningioma.

[^68^Ga]-DOTATATE is a positron emission tomography (PET) radiotracer that targets somatostatin receptor 2 (SSTR2), which is highly expressed in meningiomas, with greater affinity than other somatostatin receptor analogs^8,9^. Since achieving the orphan drug status in 2014 by the Food and Drug Administration, [^68^Ga]-DOTATATE PET has proven to be superior to other functional imaging modalities in meningioma such as Indium-111-Octreotide scintigraphic imaging, demonstrating improved specificity and target-to-background ratio^10^. [^68^Ga]-DOTATATE PET has also demonstrated its superior clinical utility in target volume delineation during radiation planning, in the detection of transosseous, small, or difficultly located meningiomas, and in post-surgical settings, when compared to contrast enhanced MRI alone^4–7,11^.

However, while highly specific to meningioma, the level of [^68^Ga]-DOTATATE avidity in meningioma that constitutes the optimal diagnostic threshold has not been systematically investigated. Furthermore, the specificity of [^68^Ga]-DOTATATE avidity in meningioma is affected by the variable physiologic uptake in other tissues including the pituitary, salivary, thyroid glands, liver, spleen, and urinary tract^12^. Several different methods have been utilized in previous studies to classify lesions as meningioma on the basis of [^68^Ga]-DOTATATE PET when interpreting standard uptake value (SUV) in meningioma, often with reference to SUV in a background tissue, such as contralateral brain parenchyma, contralateral subarachnoid space, liver, gluteal muscle, and superior sagittal sinus^4,5,13–15^. While the liver reference recapitulates the Krenning score, first established on the basis of Indium-111-Octreotide scintigraphic imaging, it does not reflect the differences in receptor specificity between [^68^Ga]-DOTATATE and Octreotide. Importantly, it requires whole-body PET in addition to brain PET, which confers longer acquisition time, thereby affecting patient comfort as well as cost. Additionally, the potential for incidental findings on the whole-body MRI may lead to undue patient anxiety and unwarranted workups^16^. Thus, approaches focusing on data obtained from dedicated brain acquisition alone hold promise in optimizing the acquisition and analysis protocols while maintaining patient comfort and limiting acquisition time.

Published diagnostic approaches include an absolute maximum SUV threshold of 2.3, which demonstrated 90% sensitivity and 73% specificity for the purpose of diagnosing meningioma from tumor free tissue, which have been utilized in several other studies^5,17,18^. For surgical and radiation planning purposes, however, which demand a higher confidence level for diagnosis and delineation of a tumor, a greater specificity may be desired. Another previously published method uses the superior sagittal sinus (SSS) as a reference, given its role as cranial blood pool, with a proposed SUV ratio (SUVR) of 3 to distinguish meningioma from post-treatment change^4^. Our purpose was to compare the four different approaches to determining optimal diagnostic thresholds using [^68^Ga]-DOTATATE PET/MR in the differentiation of meningioma versus post-treatment change in the post-surgical setting: (i) the absolute maximum SUV threshold of 2.3, (ii) the SUVR to SSS with the threshold of 3, (iii) the SUVR to the normal brain parenchyma, and (iv) the SUVR to the pituitary gland, an intracranial organ with consistently high physiologic [^68^Ga]-DOTATATE avidity. We also aimed to establish the optimal threshold SUV values for each method with the greatest sensitivity and specificity.

## Methods

### Patient population

**Figure 1:**
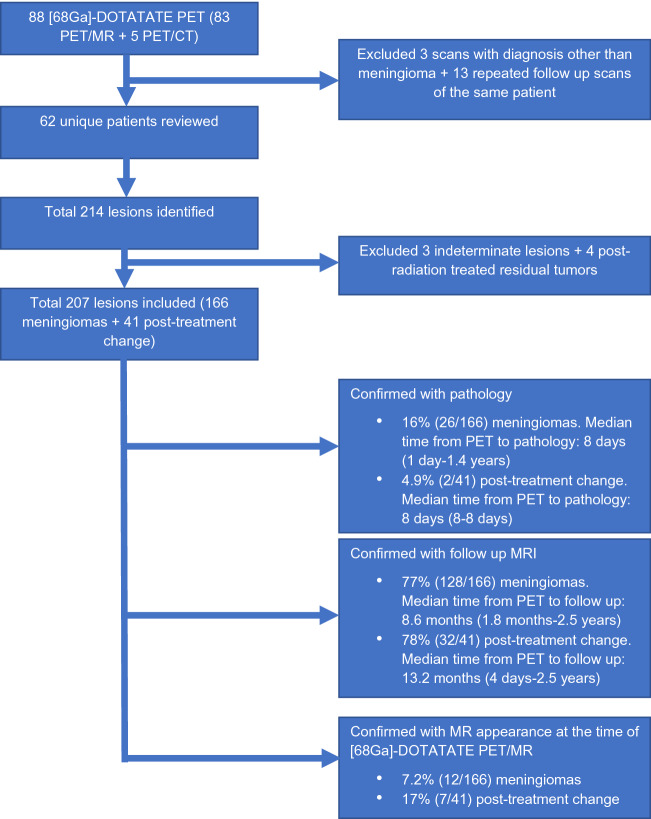
Flow chart of the included subjects and lesions as confirmed by each diagnostic criterion.

Institutional review board approval with informed consent was obtained for this retrospective HIPAA compliant study of patients with a history of clinically suspected or pathology-proven meningioma. A total of 88 [^68^Ga]-DOTATATE PET/MRI examinations were obtained between July 2018 and March 2021 as part of a prospective clinical trial (ClinicalTrials.gov Identifier: NCT04081701) for the purpose of diagnosing meningioma or differentiating recurrence from post-treatment change. Five patients who were ineligible for PET/MRI underwent PET/CT and MRI separately. From this cohort, all patients with imaging evidence of meningioma and post-treatment changes were included. All patients with a diagnosis other than meningioma were excluded (Fig. 1). In patients with multiple longitudinal [^68^Ga]-DOTATATE PET/MRI examinations, only the initial scan was included. Clinical chart review was performed to collect clinical and demographic characteristics of the study population, including age, sex, surgical history, radiation treatment history, as well as the number of meningiomas in each patient.

### Image acquisition

PET/MRI was performed on the Biograph mMR scanner (Siemens Healthineers, Erlangen, Germany) in all cases except one patient who was scanned on the GE SIGNA PET/MR scanner (GE Healthcare, Milwaukee, WI). All PET data acquisitions started at 7 ± 3 min post-injection of 172.9 ± 18.4 MBq of [^68^Ga]-DOTATATE. The PET data were continuously acquired in 3D List Mode for a total period of 50 min and then histogrammed to a single sinogram of a time frame of 7–57 ± 3 min post-injection. Although the [^68^Ga]-DOTATATE uptake in meningioma relative to background non-specific brain tissue is expected to maximize during the last 10 min of the 50-min acquisition^19^, we chose to use all 50 min of PET data, instead of the last 10 min, to minimize statistical noise in the PET data and uncertainties in the final SUV-derived scores, as past studies have shown that SUV scores from 10 to 60 min p.i. time windows are still well-differentiated between meningioma and post-treatment change regions ^4^. While typical brain PET is shorter than our 50-min acquisition time, the acquisition time of 50 min was used in our cohort for the additional, specific purpose of obtaining dynamic PET data of the tracer uptake over time, which was a focus of a different study. Given that PET and MRI are acquired simultaneously, and that our institutional brain tumor MRI protocol typically takes 45 min, the PET acquisition exceeded the MRI acquisition by only 5 min, thus minimizing additional time in the scanner for the patients.

All PET images were reconstructed with the default Ordered Subsets Expectation Maximization reconstruction algorithms of the manufacturer with Point Spread Function modeling (OSEM-PSF) using three iterations and 21 (Biograph mMR) or 28 subsets (Signa). The resulting image matrix size was 344 × 344 × 127 (192 × 192 × 89) voxels with a voxel size of 2.086 × 2.086 × 2.031 mm (1.875 × 1.875 × 2.780) mm for Biograph mMR (Signa). During image reconstruction, the PET data were corrected for attenuation, scatter, randoms, normalization, dead-time, decay and frame duration using the default settings. For attenuation and scatter correction, the manufacturer’s default method and settings for estimating the MR-based brain tissue attenuation map were employed.

MRI was performed according to institutional protocol, including pre- and postcontrast sagittal 3D T1 SPACE (TR/TE 600–700 ms/11–19 ms, 120 degree flip, 1 mm slice thickness) and postcontrast 3D T2 FLAIR (TR/TE 6300–8500 ms/394–446 ms, 120 degree flip, 1 mm slice thickness). MR-based attenuation correction was obtained according to manufacturer's standard-of-care specifications. For patients who underwent PET/CT and MRI separately, the CT image set of the PET/CT was subsequently registered to the postcontrast T1-weighted MR images using the rigid registration algorithm residing on Syngo.Via workstation (Siemens Healthineers, Erlangen, Germany) and the resulting transformation matrix was then applied to the PET image set to register it to the MRI images.

### Quantitative imaging analysis

All reconstructed PET images were initially displayed in quantitative units of Bq/mL. Then the absolute maximum SUV metric was calculated at every image voxel by dividing the respective Bq/ml pixel value with the ratio of the administered dose of the radiotracer, in units of Bq, over the subjects’ body weight (in units of g) to remove the confounding effect of radiotracer dose and body weight when quantifying the [^68^Ga]-DOTATATE uptake in every tissue. Regional absolute maximum SUV scores were subsequently extracted from a set of image pixels defining the meningioma and post-treatment change regions-of-interest (ROIs) in each PET image. As we employed the entire 50 min of acquired PET data to calculate the absolute SUV scores, we expect low noise in the PET images, thus we chose to obtain the maximum absolute SUV value at each defined ROI which is more robust to partial volume effects due to spill-out of activity concentration values from the high-uptake ROIs to surrounding low-uptake background tissue.

The resulting absolute SUV scores are considered semi-quantitative as, although they no longer depend on the subjects’ body weight and administered dose, they may still be affected by several other exam factors that could vary between scans, such as the exact scan time window position relative to injection, the actual fraction of [^68^Ga]-DOTATATE dose that initially entered the blood circulation immediately post injection and the dynamic amount that was later available in the blood stream for tissue uptake throughout the acquisition period, the physiology of the subject at the exam day which could affect [^68^Ga]-DOTATATE uptake dynamics in tissues, etc. Therefore, the comparison between absolute SUV metrics of different PET exams, even of the same subject, may be confounded by any of these factors thus limiting its quantitative value. To potentially limit these confounding effects, we normalized the ROI-based maximum absolute SUV scores with respect to the maximum absolute SUV scores of the SSS region, which in the context of meningioma and post-treatment uptake can be considered as a negative reference, the pituitary gland, which is a region always exhibiting considerably high [^68^Ga]-DOTATATE uptake, and finally the normal brain parenchyma.

The PET/MR images were read by a fellowship trained neuroradiologist with board certification in neuroradiology and nuclear medicine and another fellowship trained neuroradiologist with board certification in neuroradiology at our institution. The images were interpreted for the clinical purpose of diagnosing meningioma recurrence in the post-operative setting, and the radiologists had access to the full patient information at the time of study interpretation. Absolute SUV was primarily used given that this is, in clinical terms, a novel diagnostic approach to meningiomas that studies such as the one presented here aim to further validate. SSS and pituitary gland SUV were recorded as part of the clinical radiology report but not routinely used for clinical interpretation.

The ROIs were drawn for target lesions, including meningioma, suspected posttreatment change, SSS, the pituitary gland, and normal brain parenchyma. The lesions were confirmed as meningioma versus post-treatment change based on the pathology findings, if available, and follow up MR imaging appearance. In patients with multiple meningiomas, only the lesions that were directly biopsied or surgically removed were categorized as pathology proven. In the absence of pathology and imaging follow up, the current gold standard MRI appearance from the [^68^Ga]-DOTATATE PET/MR was used to classify the lesions. Tumors with a diameter greater than 0.6 cm and with high tracer avidity (determined visually) were included for evaluation. The anatomic delineation of the ROIs in the PET images was based on the coregistered sagittal 3D T1-weighted postcontrast MR images with respective axial and coronal reformations. MRI based classification of meningioma was based upon prototypical imaging characteristics including well-circumscribed margins, lobular morphology, avid contrast enhancement, and extra-axial location with a broad-based dural attachment, often with an associated dural tail, as determined by the interpreting neuroradiologist. Other characteristics that were considered ancillary evidence of a meningioma diagnosis included internal areas of calcification and inward displacement of adjacent cortex. The maximum SUV scores of the normal brain parenchyma were obtained from a standardized volume of 3 cc (mean volume: 3.01 cc, SD: 0.18) placed in the region of the centrum semiovale in normal-appearing (on conventional MRI) brain parenchyma contralateral to the side with most meningioma lesions in each patient.

Any indeterminate lesions were excluded. Any previously irradiated residual meningiomas were also excluded. The breakdown of the number of lesions confirmed by each of the above criteria is outlined in Fig. 1. The lesions that were confirmed as meningiomas or post-treatment changes were then reclassified using the following four methods: an absolute maximum SUV threshold of 2.3 as described in reference^17^, an SUV ratio (SUVR) referencing the superior sagittal sinus (SSS) threshold of 3 as described in reference^4^, an SUVR referencing the pituitary gland (SUVRpit), and an SUVR referencing the normal brain parenchyma (SUVRnorm). Given that no previously published threshold value references exist for the SUVRpit and SUVRnorm approaches, the optimal threshold for these methods were determined by performing Youden’s J statistics within our cohort.

### Statistical analysis

Mann–Whitney test was performed to compare SUV, SUVRsss, SUVRpit, and SUVRnorm values between meningioma and post-treatment changes. Kruskal–Wallis test was performed to compare SUV, SUVRsss,SUVRpit, and SUVRnorm values between WHO grades in meningioma. In patients with multiple meningiomas, given that many simultaneously occurring meningiomas show a uniform histology, all meningiomas, including those that were not directly biopsied, were assigned the same WHO grade within a given patient, following the methodology applied in prior studies^20–22^. In order to evaluate potential bias when analyzing multiple meningiomas per patient, an additional evaluation with only pathology proven meningiomas was performed. Linear regression analysis was performed to correlate SUV, SUVRsss, SUVRpit, and SUVRnorm with the size of meningioma as determined by the length of the longest dimension in a subset of patients whose meningioma size information was available. Diagnostic accuracy of the four different classification methods was assessed using 2 × 2 contingency tables and sensitivity, specificity, positive predictive value (PPV), and negative predictive value (NPV) were calculated for each method. In order to assess PPV and NPV as directly measured in our clinical cohort, the prevalence of meningioma in our cohort (80.2% (166/207 lesions of interest)) was used to calculate PPV and NPV. The statistical difference between the four diagnostic methods was evaluated using the McNemar test. Receiver-operating-characteristic analyses were performed to compare the diagnostic performance of SUV, SUVRsss, SUVRpit, and SUVRnorm using area under the curve. Youden’s J statistics was performed to identify the optimal thresholds in each method. At the identified optimal thresholds, the McNemar test was again used to compare the four diagnostic methods. GraphPad Prism 8 was used to perform all statistical analyses. P values below 0.05 were considered to indicate statistical significance.

### Ethics approval and consent to participate

Institutional review board (IRB) approval was obtained from the IRB committee of Weill Cornell Medicine (WCM) for this HIPAA compliant study. All experimental protocols were approved by the WCM IRB committee. Informed consent was obtained from all subjects. All methods were carried out in accordance with relevant guidelines and regulations.

### Consent for publication

Consent to publish any individual data has been obtained as part of the informed consent process.

## Results

### Study population

Of the initial cohort of 88 [^68^Ga]-DOTATATE PET/MRI and PET/CT, 3 scans with diagnosis other than meningioma and 13 scans that were repeated follow up scans of the same patient were excluded. In total, 62 patients met the inclusion criteria (Fig. 1). Among the 214 lesions identified on [^68^Ga]-DOTATATE PET/MR in 62 patients, 3 indeterminate lesions in the absence of pathology and imaging follow ups and 4 lesions that were post-radiation treated residual tumors were excluded. In total, 166 meningiomas and 41 post-treatment change lesions were identified across the cohort. Of the 166 meningiomas, 16% (26/166) were confirmed with pathology outcome, with the median time from PET to pathology of 8 days (range: 1 day–1.4 years) (Fig. 1). 4.9% (2/41) of the post treatment change lesions were confirmed with pathology, with median time from PET to pathology of 8 days. 77% (128/166) of meningiomas and 78% (32/41) of post-treatment change were confirmed with follow up MRI, with the median time from PET to follow up of 8.6 months for meningioma (1.8 months–2.5 years) and of 13.2 months for post-treatment change (4 days–2.5 years). Finally, 7.2% (12/166) of meningiomas and 17% (7/41) of post treatment change were confirmed on the basis of the MR appearance at the time of [^68^Ga]-DOTATATE PET/MR. Detailed clinical and demographic characteristics of the study population are outlined in Table 1. A representative patient images from the cohort is shown in Fig. 2.

**Table 1:** Demographic characteristics of the study population.

# Patients	62
# Patients with a history of pathology proven meningioma	87% (54/62)
Age	55.9 (21–89)
Sex	66% F (41/62)
Surgical history	85% (53/62)
Time from surgery to PET	Median: 8.0 months (19 days–14.6 yrs)
Radiation history	23% (14/62)
Time from radiation to PET	Median: 27.6 months (10 months–17.3 yrs)
N meningiomas	166
N post-treatment changes	41
N meningiomas per patient	15% (9/62) with 0 meningioma
	35% (22/62) with 1 meningioma
	34% (21/62) with 2–4 meningiomas
	16% (10/62) with > 4 meningiomas
	Median: 1.5 meningiomas per scan (0–16)
N post-treatment change lesions per patient	53% (33/62) with 0 post-tx change
	32% (20/62) with 1 post-tx change
	15% (9/62) with 2–4 post-tx changes
	Median: 0 post-tx change per scan (0–4)
WHO grade	31% (19/62) WHO grade 1
	47% (29/62) WHO grade 2
	10% (6/62) WHO grade 3
	13% (8/62) WHO grade not available

**Figure 2:**
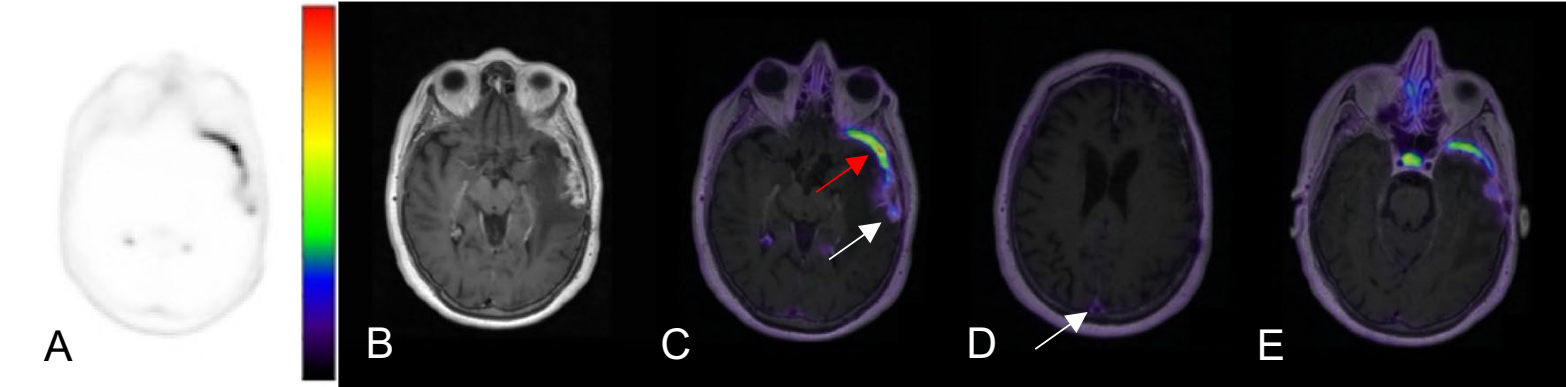
Axial images of [^68^Ga]-DOTATATE PET AC (**A**), 3D T1 post-gadolinium MR (**B**), fused PET/MR T1 (**C**–**E**) windowed SUV 0–15. This representative patient from the cohort is a 70-year-old female with a history of WHO grade II meningioma s/p resection and proton therapy 6 years prior to imaging and an additional proton therapy a year prior. The fused PET/MR images demonstrate a lesion suspicious for a meningioma in the left anterior temporal pole with SUV of 13.6 (**C**, red arrow). The more posteriorly located enhancing lesion (**C**, white arrow) demonstrates SUV of 4.5 and was suspicious for post-treatment change, given that the SUV of the superior sagittal sinus was 2.3. The subsequent resection and biopsy of the two lesions a year later confirmed the suspected diagnosis of recurrent meningioma and radiation necrosis, respectively. The superior sagittal sinus (**D**, arrow) demonstrates SUV of 2.3 and the pituitary gland (**E**) demonstrates SUV of 12.4.

### Descriptive and correlative analysis of SUV

**Figure 3:**
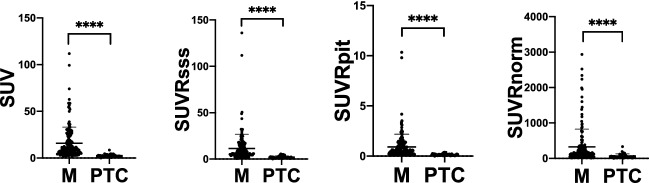
Meningioma (M) versus Post-treatment change (PTC) SUV, SUVRsss, SUVRpit, and SUVRnorm with mean and standard deviations. Mean SUV, SUVRsss, SUVRpit, and SUVRnorm of meningioma was 15.8, 11.5, 0.92, and 324.7 respectively. **** indicates statistical significance with p < 0.0001 as determined by Mann–Whitney test.

**Figure 4:**
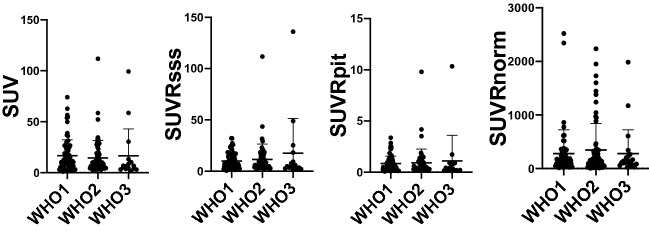
Meningioma SUV, SUVRsss, SUVRpit, and SUVRnorm stratified by WHO Grades with mean and standard deviations. Kruskal–Wallis test showed no statistically significant relationship between WHO Grade and the four SUV metrics.

Mean and range of SUV in meningioma and post-treatment change lesions as well as in the pituitary gland and SSS of the cohort are outlined in Table 2. Mean SUV of meningioma was significantly higher than that of post-treatment change lesions (15.8 vs. 2.58, P < 0.0001) (Fig. 3). Of note, the SUV range of normal brain parenchyma was largest relative to its mean SUV value, compared to the pituitary gland and SSS. Youdon’s J statistics revealed 0.3 and 62.6 as the optimal thresholds for SUVRpit and SUVRnorm, respectively. Mean SUVRsss, SUVRpit, and SUVRnorm of meningioma were also significantly higher than that of post-treatment change lesions (11.5 vs. 2.10, P < 0.0001; 0.92 vs. 0.16, P < 0.0001; 324.7 vs. 64.96, P < 0.0001, respectively) (Fig. 3). There was no correlation between WHO grade and SUV, SUVRsss, SUVRpit, or SUVRnorm of meningioma in the cohort (P = 0.23, P = 0.56, P = 0.23, P = 0.88, respectively) (Fig. 4). The additional analysis with the lesions that are pathology proven confirmed this relationship. Regression analysis revealed lesion size as a significant predictor of all SUV, SUVRsss, SUVRpit, and SUVRnorm in meningioma (P < 0.005, R2 = 0.066; P < 0.0005, R2 = 0.096; P = 0.0014, R2 = 0.079; P < 0.0001, R2 = 0.124, respectively).

**Table 2:** Descriptive analysis of the target lesions.

	Mean (range)
Pituitary SUV	16.7 (7–34.2)
SSS SUV	1.39 (0.6–2.8)
Normal brain parenchyma SUV	0.0725 (0.01–0.4)
Normal brain parenchyma ROI volume	3.01 (2.45–3.31)
Meningioma SUV	15.8 (1.1–111.8)
Meningioma SUVRsss	11.5 (0.52–136.1)
Meningioma SUVRpit	0.92 (0.065–10.3)
Meningioma SUVRnorm	324.7 (6.75–2935)
Meningioma size (n = 126) (cm)	1.34 (0.2–4.7)
Post-Tx change SUV	2.58 (0–8.5)
Post-Tx change SUVRsss	2.1 (0–5.5)
Post-Tx change SUVRpit	0.16 (0–0.39)
Post-Tx change SUVRnorm	64.96 (0–330)
Post-Tx Change Size (n = 9) (cm)	0.9 (0.3–1.6)

Only a subset (n = 9) of the post-treatment change lesions had its size information available.

### Diagnostic performance of the four classification methods at the pre-determined thresholds

Contingency tables for the four classification methods are shown in Tables 3, 4, 5, and 6. Sensitivity, specificity, PPV, and NPV of the four methods are compared in Table 7, along with the prevalence of meningioma in the cohort. At the pre-determined thresholds, SUV demonstrated the highest sensitivity (98.2%) and the highest specificity was achieved with SUVRpit (87.8%) (Table 7). As shown in Table 8, the sensitivity of the SUV method was significantly greater than the sensitivities of both SUVRsss and SUVRpit methods (P < 0.0001). However, the specificity of the SUV method was significantly lower than that of both SUVRsss and SUVRpit (P = 0.0044 and P = 0.0009, respectively). The sensitivity of SUVRsss was significantly higher than that of SUVRpit (P = 0.006) while the specificities of both SUVR methods did not differ significantly (P = 0.3711). The SUVRnorm method did not differ significantly from the SUVRpit method, while its sensitivity was significantly lower than that of SUVRsss method (P = 0.0371).

**Table 3:** Contingency table for the SUV method at the pre-determined threshold of 2.3.

> 2.3 SUV	Meningioma	Post-Tx change	Total
Positive	163	18	181
Negative	3	23	26
Total	166	41	207

**Table 4:** Contingency table for the SUVRpit method at the optimal threshold of 0.3.

> 0.3 SUVRpit	Meningioma	Post-Tx Change	Total
Positive	132	5	137
Negative	34	36	70
Total	166	41	207

**Table 5:** Contingency table for the SUVRsss method at the pre-determined threshold of 3.

> 3 SUVRsss	Meningioma	Post-Tx Change	Total
Positive	144	8	152
Negative	22	33	55
Total	166	41	207

**Table 6:** Contingency table for the SUVRnorm method at the optimal threshold of 62.6.

> 62.6 SUVRnorm	Meningioma	Post-Tx Change	Total
Positive	134	12	146
Negative	32	29	61
Total	166	41	207

**Table 7:** Sensitivities, specificities, positive predictive value (PPV), negative predictive value (NPV) of the four methods with 95% confidence interval at the pre-determined thresholds.

	> 2.3 SUVmax	> 0.3 SUVRpit	> 3 SUVRsss	> 62.6 SUVRnorm
Sensitivity	98.2% (94.8–99.5)	79.5% (72.7–85.0)	86.7% (80.8–91.1)	80.6% (73.9–85.9)
Specificity	56.1% (41.0–70.1)	87.8% (74.5–94.7)	80.5% (66.0–89.8)	70.7% (55.5–82.4)
PPV	90.1% (84.8–93.6)	96.4% (91.7–98.4)	94.7% (90.0–97.3)	91.7% (86.1–95.2)
NPV	88.5% (71.0–96.0)	51.4% (40.0–62.8)	60.0% (46.8–71.9)	47.5% (35.5–59.8)
Prevalence	80.2% (166/207)	80.2% (166/207)	80.2% (166/207)	80.2% (166/207)

**Table 8:** McNemar’s Test Results comparing the four methods at the pre-determined thresholds.

	SUV vs SUVRpit	SUVRpit vs SUVRsss	SUV vs SUVRsss	SUV vs SUVRnorm	SUVpit vs SUVRnorm	SUVRsss vs SUVRnorm
Sensitivity	P < 0.0001*	P = 0.0060*	P < 0.0001*	P < 0.0001*	P = 0.8445	P = 0.0371*
Specificity	P = 0.0009*	P = 0.3711	P = 0.0044*	P = 0.0412*	P = 0.0961	P = 0.2888

### Diagnostic performance of the four classification methods at their optimal thresholds

**Figure 5:**
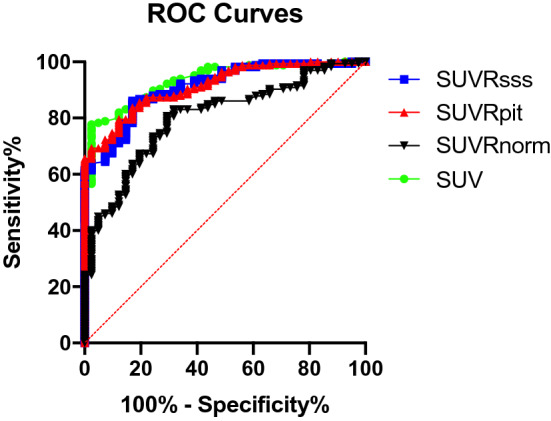
ROC Curves of the four methods: SUV, SUVRsss, SUVRpit, and SUVRnorm. ROC analysis revealed area under the curve of 0.932, 0.910, 0.915, and 0.800 for SUV, SUVRsss, SUVRpit, and SUVRnorm, respectively (all P < 0.0001).

Table 9 shows the optimal thresholds for each of the four methods as determined by Youden’s J statistics and the sensitivities and specificities for each method at the optimal thresholds. Of note, the optimal threshold for SUVRsss method was found to be 3.2, similar to the threshold of 3 as used in the previous study^4^. The optimal threshold for SUV was 4.7 in our cohort, greater than the pre-determined threshold of 2.3. At the optimal thresholds, SUV had the highest specificity (97.6%) and SUVRsss had the highest sensitivity (86.1%). The sensitivity of SUV was significantly lower than that of SUVRsss while the specificity of SUV was significantly higher than that of SUVRsss (P = 0.0012 and P = 0.0412, respectively) (Table 10). The SUVRpit method was not statistically different from the SUV method in both sensitivity and specificity, however it had a significantly lower sensitivity than the SUVRsss method (P = 0.0098) with no significant difference in specificities. The SUVRnorm method did not significantly differ from the other three methods in terms of sensitivity, but its specificity was significantly lower than that of the SUV method (P = 0.0055). A receiver-operating-characteristic analysis of SUV, SUVRsss, SUVRpit, and SUVRnorm as diagnostic parameters for meningioma revealed area under curve of 0.932 (P < 0.0001), 0.910 (P < 0.0001), 0.915 (P < 0.0001), and 0.800 (P < 0.0001), respectively (Fig. 5).

**Table 9:** Optimal threshold of each method as determined by Youden’s J Statistics with 95% confidence interval.

Methods	J	Optimal Threshold	Sensitivity	Specificity
SUV	75.27	> 4.7	77.7% (70.8–83.4)	97.6% (87.4–99.9)
SUVRsss	69.07	> 3.2	86.1% (80.1–90.6)	82.9% (68.7–91.5)
SUVRpituitary	67.32	> 0.3	79.5% (72.7–85.0)	87.8% (74.5–94.7)
SUVRnorm	51.34	> 62.6	80.6% (73.9–85.9)	70.7% (55.5–82.4)

**Table 10:** The McNemar’s Test for sensitivity/specificity of the four methods at the optimal thresholds.

	SUV vs SUVRpit	SUVRpit vs SUVRsss	SUV vs SUVRsss	SUV vs SUVRnorm	SUVpit vs SUVRnorm	SUVRsss vs SUVRnorm
Sensitivity	P = 0.505	P = 0.0098*	P = 0.0012*	P = 0.5563	P = 0.8445	P = 0.0662
Specificity	P = 0.1336	P = 0.6171	P = 0.0412*	P = 0.0055*	P = 0.0961	P = 0.1824

## Discussion

Recurrence is relatively common in meningioma patients post-treatment, occurring in up to 20% of cases, even in histologically benign (WHO grade 1) cases^23^. In order to minimize the rate of recurrence as well as the side effects from excessive treatment to normal brain tissue, an ideal imaging modality should be specific enough to define the correct tumor volume from tumor-free tissue, thereby aiding in surgical and radiation planning to achieve the maximal safe target volume. In the post-treatment setting, it should also be sensitive to detect any residual or recurrent tumor from post-treatment scarring and inflammation. The most recently updated European Association of Neuro-Oncology guideline for the management of meningioma continues to highlight contrast-enhanced MRI as the gold standard for diagnosing and follow up imaging in meningioma patients^24^. However, MRI appearance is often limited in accuracy particularly in the presence of post-treatment scarring and inflammation. Rachinger and colleagues demonstrated that standard MRI has sensitivity of 79% and specificity of 65% in distinguishing meningioma from tumor-free tissues^17^. Another group showed that MRI alone can achieve sensitivity of 95% and specificity 88% in the diagnosis of meningioma in a cohort of 57 patients, but noted that diagnostic accuracy diminishes in cases of small lesions (< 0.5 cm^3^) and certain difficult locations such as skull base^6^. For transosseous growing meningiomas, MRI’s sensitivity was even lower at 54%^5^.

The recently updated European Association of Neuro-Oncology guidelines also highlight the role of [^68^Ga]-DOTATATE PET in distinguishing meningioma from healthy tissue and post-surgical changes^24^. Histology-controlled studies showed that the extent of meningiomas is better delineated with [^68^Ga]-DOTATATE PET compared to contrast-enhanced MRI alone^5,17^. During radiation planning, [^68^Ga]-DOTATATE and DOTATOC PET alter target volume delineation for stereotactic fractionated radiation therapy, often resulting in a reduction of the gross tumor volume compared with results from MRI or CT^11,25,26^. The utility of adjuvant RT, compared to active surveillance, in resected meningiomas is currently being evaluated in the NRG0539 trial, in which our preliminary analysis suggests [^68^Ga]-DOTATATE’s efficacy for RT response assessment with a marked reduction in [^68^Ga]-DOTATATE SUV in meningioma post-RT. Additionally, a case series of 20 patients demonstrated the clinical utility of [^68^Ga]-DOTATATE PET/MR in identifying additional meningiomas not previously identified on contrast-enhanced MRI and in differentiating disease from reactive enhancement, thus facilitating treatment planning in such cases^4^. Further reinforcing its clinical utility, [^68^Ga]-DOTATATE PET has shown efficacy in predicting progression in non-benign meningioma as well as predicting clinical outcome for SSTR targeted radionuclide therapy such as Lu-DOTATATE^18,20^. Within our cohort, we confirmed that all four approaches to [^68^Ga]-DOTATATE PET quantification (absolute SUV, SUVRsss, SUVRpit, and SUVRnorm) aid in the differentiation of meningioma from post-treatment changes, confirming the reliability of the SSTR2 targeted imaging in meningioma patients. SUV, SUVRsss, SUVRpit, and SUVRnorm did not correlate with WHO grade, consistent with prior histopathological studies, and suggesting that SSTR2 expression is independent of the differentiation status of meningioma tumor cells^17,27^. There was a significant relationship observed between tumor size and SUV, SUVRsss, SUVRpit, and SUVRnorm.

In order to effectively utilize [^68^Ga]-DOTATATE PET in the clinical context of meningioma, an SUV threshold that constitutes as the diagnostic threshold must be established. Prior studies often employed reference tissue SUVR approaches, such as contralateral brain parenchyma, contralateral subarachnoid space, liver, gluteal muscle, and superior sagittal sinus^4,5,13–15^. One prospective study of 21 patients reported absolute SUV threshold of 2.3 with 90% sensitivity and 73% specificity for the purpose of diagnosing meningioma from tumor free tissue, which have been utilized in several other studies^5,17,18^. One other well-known method is the Krenning score system, derived from [111In]Octreotide scintigraphy of gastrointestinal neuroendocrine tumors, which uses the liver and spleen as the reference regions and has been validated in neuroendocrine tumors that are SSTR2 positive for the purpose of assessing candidacy for PRRT^28^. However, the Krenning score has not been utilized in meningioma and requires a body PET. Therefore, we evaluated PET/MRI-based approaches to quantitative [^68^Ga]-DOTATATE PET analysis that do not require whole-body imaging, and instead rely on dynamic brain PET imaging. Amongst the anatomic regions that can be obtained from brain PET, we chose the SSS or cranial blood pool as a background reference region, as previously published, the pituitary gland, a notably SSTR2 positive intracranial organ, and the normal brain parenchyma^4^.

In our cohort of 62 patients, at the pre-determined threshold, the SUV method with the threshold of 2.3 demonstrated the highest sensitivity (98.2%) but much lower specificity than the prior study of 21 patients (56.1% vs. 73.5%)^5^. The optimal threshold for SUV in our cohort was 4.7, much greater than the threshold of 2.3 in the prior study^5^. This discrepancy may be explained by the different sample sizes, varying acquisition time and technicality of imaging, and random variation in SUV across patients. The highest specificity was achieved with SUVRpit (87.8%) with the threshold of 0.3 but it was not significantly different from the specificity of SUVRsss at the pre-determined threshold of 3, which had a greater sensitivity than SUVRpit. We then recalculated sensitivity and specificity of each classification method at the optimal threshold as determined by Youden’s J statistics. Notably, the optimal threshold for SUVRsss was 3.2, similar to 3 as set by the prior study and as tested in our analysis of the pre-determined thresholds^4^. Interestingly, at the optimal thresholds, the methods with the highest sensitivities and specificities were flipped; SUV had the highest specificity (97.6%) and SUVRsss had the highest sensitivity (86.1%). At the optimal thresholds, the SUVRpit method was not statistically different from the SUV method, however it had a significantly lower sensitivity than the SUVRsss method with no difference in specificities, suggesting that SUVRsss is a superior method overall compared to SUVRpit. While the SUVRnorm method did not significantly differ from the other three methods in terms of sensitivity, its specificity was the lowest of the four methods (70.7%) which was significantly lower than that of the SUV method (P = 0.0055). Given that the AUC of the ROC for the SUVRnorm method was the smallest of the four methods, combined with the fact that the normal brain parenchyma measurements had the largest range of SUV values relative to its mean SUV value, we conclude that the SUVRnorm method is the least robust method in our cohort. AUCs of the ROC for the other three methods were comparable. Based on this result, we conclude that SUVRsss with the threshold of 3.2 may be used in clinical settings where greater sensitivity is desired such as in the post treatment setting in the evaluation of recurrence or progression. The SUV threshold of 4.7 may be more appropriate for instances where high specificity is desired such as surgical and radiation planning. However, it is important to note that SUVR that is normalized to a region of interest in the same patient may be more reproducible and thus a more reliable metric of SSTR expression, allowing more robust comparison across time points, scanners, and patients. Furthermore, given greater variability of SUV of the pituitary gland compared to SUV of the SSS, SUVRsss may be the more robust of the two SUVR methods and thus the preferred method in the clinical setting.

Our study has several limitations. It is important to note that a significant number of individual lesions included in the study (77% of meningiomas) were confirmed based on clinical follow ups rather than biopsy. For the purpose of correlating WHO grades and SUV in meningioma, multiple meningiomas in a given patient were assigned the same WHO grade as the WHO grade of the pathology proven meningioma in the given patient, as applied in previously published studies^20–22^. Additionally, the number of the pathology proven post-treatment change is relatively low given that they are most often confirmed with longitudinal follow ups with MRI as the gold standard. Notably, 17% of the post-treatment change lesions included in the study were confirmed with the MR appearance of the [^68^Ga]-DOTATATE PET/MR in the absence of both biopsy and clinical follow ups, which may affect the accuracy of the classification. Additionally, while we excluded meningiomas that were previously irradiated from our analysis, recurrence in prior RT fields might affect SUV of the lesions when compared with de novo or untreated meningiomas, an important issue which we plan to study in future work. Finally, a SSTR2 negative meningioma, although exceedingly rare, may contribute to potential misclassification of the lesions^29^.

## Conclusion

[^68^Ga]-DOTATATE PET/MR has emerged in recent years as a useful adjunct modality for management of meningioma in various clinical contexts, including diagnosis, treatment planning and response assessment, and recurrence surveillance. Our study represents a systematic comparison of quantitative analysis approaches to differentiating meningioma from post-treatment change. We compared absolute SUV as well as SUVR thresholds in their diagnostic performance in diagnosing meningioma in the post treatment setting and determined the optimal numerical threshold values that achieve the greatest sensitivity and specificity. Our analysis reveals that the SUVRsss approach with an optimal threshold of 3.2 achieves the greatest sensitivity while the absolute SUV threshold of 4.7 has the highest specificity. Recognizing that the SUVR method may be a more reproducible metric, the SUVRsss method may convey the greatest clinical utility. Further studies that investigate our threshold values in cohorts of varying sizes and compositions are required to validate our findings in other clinical contexts and assess the effect of demographic factors on SSTR biology in meningioma. Continued efforts to standardize interpretation and diagnostic criteria of [^68^Ga]-DOTATATE PET/MR imaging in meningioma have the potential of improving diagnosis and treatment and thereby improve clinical outcomes for patients with meningioma.

## Data Availability

The datasets used and/or analyzed during the current study are derived from an ongoing clinical trial and are available from the corresponding author on reasonable request.

## Abbreviations

PFS: Progression free survival
OS: Overall survival
RT: Radiation treatment
MRI: Magnetic resonance imaging
SSTR2: Somatostatin receptor 2
PET: Positron emission tomography
SUV: Standard uptake value
SSS: Superior sagittal sinus
SUVR: Standard uptake value ratio
ROI: Region of interest
SUVRpit: Standard uptake value ratio relative to pituitary gland
SUVRsss: Standard uptake value ratio relative to superior sagittal sinus
SUVRnorm: Standard uptake value ratio relative to normal brain parenchyma
PPV: Positive predictive value
NPV: Negative predictive value
WHO: World Health Organization
